# Whole-cell catalysis by surface display of fluorinase on *Escherichia coli* using N-terminal domain of ice nucleation protein

**DOI:** 10.1186/s12934-021-01697-x

**Published:** 2021-10-29

**Authors:** Xinming Feng, Miaomiao Jin, Wei Huang, Wei Liu, Mo Xian

**Affiliations:** 1 CAS Key Laboratory of Biobased Materials, Qingdao Institute of Bioenergy and Bioprocess Technology, Chinese Academy of Sciences, Qingdao, China; 2 University of Chinese Academy of Sciences, Beijing, China

## Abstract

**Background:**

Fluorinases play a unique role in the production of fluorine-containing organic molecules by biological methods. Whole-cell catalysis is a better choice in the large-scale fermentation processes, and over 60% of industrial biocatalysis uses this method. However, the in vivo catalytic efficiency of fluorinases is stuck with the mass transfer of the substrates.

**Results:**

A gene sequence encoding a protein with fluorinase function was fused to the N-terminal of ice nucleation protein, and the fused fluorinase was expressed in *Escherichia coli* BL21(DE3) cells. SDS-PAGE and immunofluorescence microscopy were used to demonstrate the surface localization of the fusion protein. The fluorinase displayed on the surface showed good stability while retaining the catalytic activity. The engineered *E.coli* with surface-displayed fluorinase could be cultured to obtain a larger cell density, which was beneficial for industrial application. And 55% yield of 5′-fluorodeoxyadenosine (5′-FDA) from S-adenosyl-L-methionine (SAM) was achieved by using the whole-cell catalyst.

**Conclusions:**

Here, we created the fluorinase-containing surface display system on *E.coli* cells for the first time. The fluorinase was successfully displayed on the surface of *E.coli* and maintained its catalytic activity. The surface display provides a new solution for the industrial application of biological fluorination.

**Graphical Abstract:**

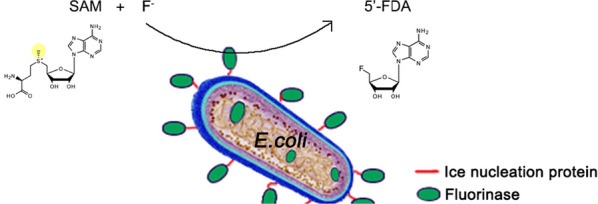

**Supplementary Information:**

The online version contains supplementary material available at 10.1186/s12934-021-01697-x.

## Introduction

Fluorine is a very special element because of its unique properties, such as the strong electronegativity, small atomic radius and so on. The van der Waals radius of fluorine is close to hydrogen, which makes fluorine substitutions of hydrogen reasonable in space structure [1]. The C-F bond is one of the strongest chemical bonds, which makes organic fluorides tend to be biologically inert [2]. In addition, fluorinated chemicals are more lipophilic than their nonfluorinated counterparts [3]. Due to these particularities, fluorine has been used to modify the physical and chemical properties of compounds, and has attracted increasingly attention in pharmaceuticals, chemicals and materials fields [4].

Fluorine is the most common halogen element in the earth crust. However, due to the low bioavailability of fluorine in nature, only a few natural organic fluorides were identified. Chemical methods have been developed to introduce fluorine into organic compounds, which often use toxic fluorination reagents [5]. In contrast, fluorinases offer a more environment-friendly approach to incorporating fluoride ions into organic compounds. Since the first fluorinase which mediate the conversion of SAM and fluoride ions into 5′-FDA was reported in 2002, there have been some reports on application cases [6]. [18F]-radiolabelled products, which was preferred for Positron Emission Tomography imaging, was synthesized with the participation of fluorinase [7, 8]. Despite the tremendous potential of industrial application, there is still a lot of work to be done before fluorinases can be applied on an industrial scale. At present, some researches have been done to improve activity and stability of fluorinase [9–11]. Immobilization of the fluorinase was carried out to improve the stability of the enzyme, meanwhile, to make it easier to remove the catalyst [9, 12, 13]. However, the process of immobilization was also accompanied by the tedious purification steps of the enzyme. By contrast, cell fermentation is more effective in large-scale applications. Previous studies have found that two shortcomings have severely limited catalytic fluorination in vivo. On the one hand, the transport of SAM to the cell interior was blocked by cell membrane [14]. On the other hand, the intracellular fluoride concentration in wild-type *E. coli* was very low because of a CrcB channel protein, which was responsible for the excretion of fluoride ions [15]. To solve the difficulty that the substrate can not be enriched in vivo, Markakis et al. constructed the engineered *E. coli* strain by adding a SAM transporter and eliminating fluoride efflux capacity and achieved fermentation production [16]. Although in vivo fluorination of *E. coli* has been achieved, the engineered cells were more sensitive to toxic fluoride ions [17]. In addition, the 5′-FDA product was accumulated inside the cell, which may cause trouble for later product extraction [16]. By the way, enzymes inside the cells can not be reused as extraction of the product requires cell disruption. The industrial application of fluorinase needs more exploration.

Surface display technology allows peptides and small proteins to be located on the cell surface and has been widely applied in live vaccines, whole-cell biocatalysts, biodetoxification, peptide library screening and biosensors [18–21]. Since the protein is outside the cell which could avoid the restriction of substrates transport across the membrane, it is suggested that the biofluorination can be combined with surface display technology. However, the research of fluorinases displayed on the cell surface has not been reported. It is also challenging to obtain an active fusion enzyme. For example, Gustavsson et al. reported an inactive surface expression of ω-transaminase, because the dimer structure was difficult to form on the cell surface [22]. Whether the hexamer structure of fluorinase can be successfully displayed on the cell surface requires further research [23].

The ice nucleation proteins are membrane proteins commonly found in genera *Pseudomonas, Panteola (Erwinia), and Xanthomonas*, which mediates the formation of ice nucleus for ice growth [24]. The protein sequence can be divided into N-terminal, C-terminal and relatively conserved central domain. The N-terminal domain which containing 170 amino acid residues has been used to display target proteins on the cell surface and showed excellent biocatalysis [21, 25]. Herein, a fluorinase mutant (Faa) from *Amycolatopsis sp.* CA-128772 was first displayed on *Escherichia coli* using the N-terminal domain of ice nucleation protein, and then whole-cell transformation was carried out to obtain 5′-FDA. To our knowledge, this is the first application of surface display technology for biological fluorination.

### Results and discussion

### Location of fluorinase in pET28a-INP-Faa-Expressing Cells

**Fig. 1:**
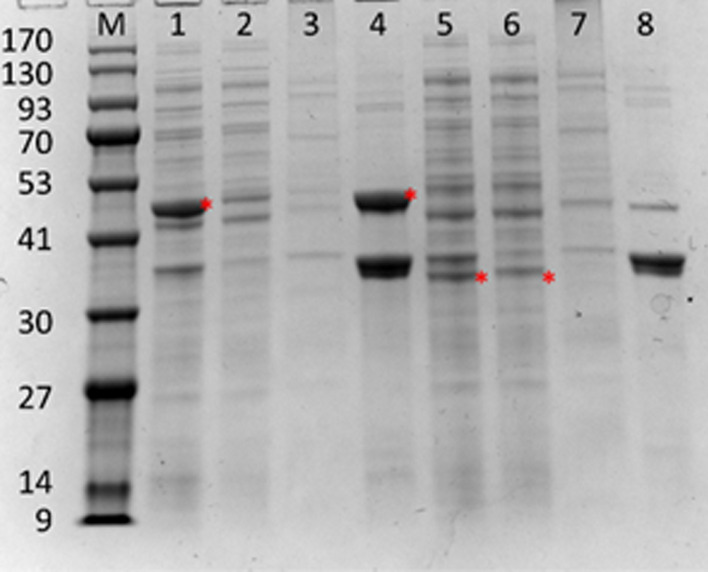
Expression of INP-Faa in different fractions of the induced cell. Lane 1: Total protein components of INP-Faa; lane 2: cytoplasmic proteins of INP-Faa; lane 3: inner membrane of INP-Faa; lane 4: outer membrane of INP-Faa; lane 5: Total protein components of Faa; lane 6: cytoplasmic proteins of Faa; lane 7 inner membrane of Faa; lane 8: outer membrane of Faa. The corresponding target protein was marked in red

The N-terminal domain of ice nucleation protein (INP) from *Pseudomonas borealis* was linked with Faa to create INP-Faa by genetic engineering. To investigate the expression location of INP-Faa in the *E.coli* cells, the protein from cytoplasm, inner membrane and outer membrane fractions were verified by SDS-PAGE (Fig. 1). The theoretical molecular weight of INP-Faa was about 50 kDa, while that of Faa was about 32 kDa. The corresponding bands could be identified in the total protein section, which meant that the proteins were expressed normally. In contrast to the control group (Faa), the 50 kDa band related to the INP-Faa was clearly observed in the lane of outer membrane fractions. This result indicated that the fluorinase can be displayed on the surface of *E.coli* cells using the N-terminal of ice nucleation protein.

The full-length ice nucleation protein, the N-terminal domain and a fusion region between N-terminal and C-terminal have been successfully applied to the surface display of C-terminal fusion partners [26]. Since the full-length fusion protein between ice nucleation protein and fluorinase is quite large, the functional expression of the fusion protein may be affected. It seemed that the N-terminal domain of INP was a better protein carrier. Therefore, truncated ice nucleation protein was used for the surface display of fluorinase.

### Immunofluorescence microscopy

**Fig. 2:**
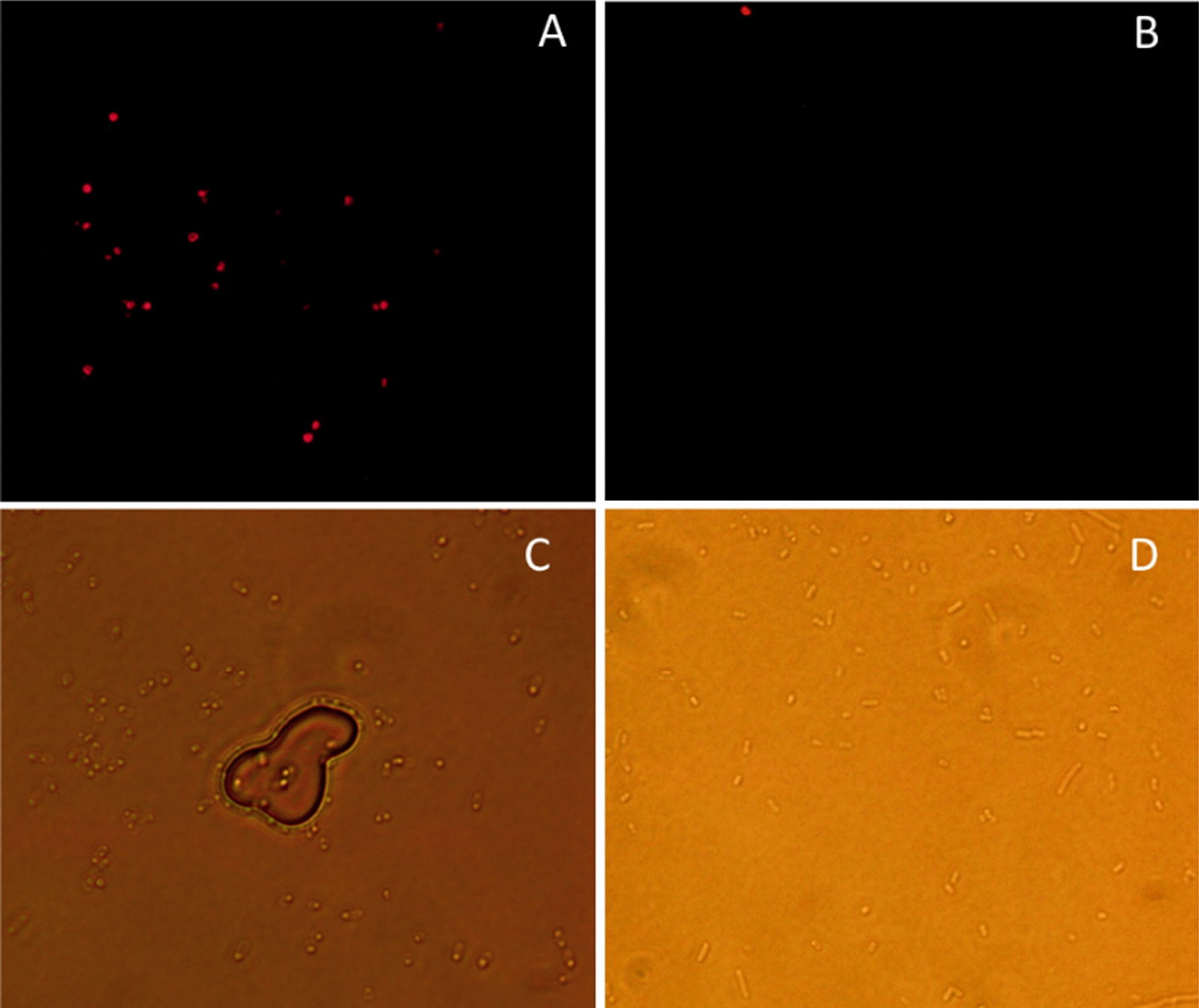
Immunoflurescence micrographs of BL21(DE3). **A** Cells harboring INP-Faa with a his-tag probed with his-tag antibody and fluorescently stained with Alexa Fluor 555-labeled Donkey Anti-Mouse IgG (H + L). **B** Cells harboring Faa with a his-tag probed with anti-his-tag and fluorescently stained with Alexa Fluor 555-labeled Donkey Anti-Mouse IgG (H + L). **C** Cells in A under regular microscope. **D** Cells in B under regular microscope

In order to confirm that INP-Faa was indeed located on the outside of the outer membrane, cell immunofluorescence assay was carried out with his-tag antibody and Alexa Fluor 555-labeled Donkey Anti-Mouse IgG (H + L). Under normal conditions, large molecules such as antibodies cannot penetrate the cell membrane, which means that only proteins on the cell surface can be stained. As expected, cells harboring the INP-Faa showed detectable fluorescence signal, indicating that the fusion protein was displayed successfully on the cell surface (Fig. 2). The small amount of fluorescence in the control (Fig. 2D) may be caused by cell death. Compared the image under regular microscope without fluorescence (Fig. 2C), not all the cells can be observed under fluorescent light (Fig. 2A). This may be related to the fact that the surface display efficiency of cells was not 100% [27].

### Whole-cell assay

**Fig. 3:**
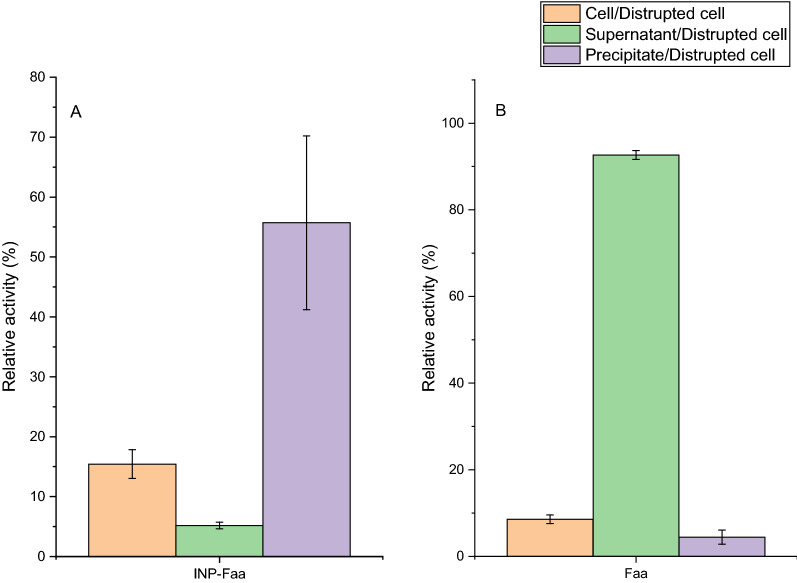
The relative activity of different fractions to the cell fragmentation. **A** BL21(DE3)/pET28a- INP-Faa. **B**: BL21(DE3)/pET28a -Faa. The assays were carried out in Tris–HCL buffer (pH 7.8, 50 mM) containing 20 mM KF, 1 mM SAM and induced cells or cell fragmentation at equal quantity for 1 h at 37 ℃. Relative values were based on catalytic ability of the cell to the disrupted cell suspension. Results are presented as the mean values and standard deviations of data from three independent biological replicates

Regardless of the normal expression and correct localization of the fusion protein, obtaining the fusion protein with activity is also affected by the secondary structure. The crystal structure of wild type fluorinase from *S. cattleya* has been resolved by Dong et.al, and the structure revealed a hexamer composed of two trimeric units [23]. Thus, it is a challenge to obtain active INP-Faa on the cell surface. The interesting thing was that the fluorinase activity of INP-Faa can be measured by HPLC (Fig. 3). For Faa cells, the catalytic activity of whole-cell was lower than that of disrupted cell suspension, which means that the permeability of cell membrane does limit the catalytic ability of whole-cell. Since the protein expression intensity of different cells is different, it may affect the ability of the apparent whole-cell catalysis. To verify the contribution of surface display enzymes to whole-cell catalysis, the disrupted cell suspension which contained all enzymes expressed in the cell was set as a relative activity of 100%. For INP-Faa cells, the majority of activity was detected in the precipitate. This result indicated that most of the target protein has been localized in the cell membrane, and after fragmentation, it formed a precipitate along with large cell debris. Compared with the intracellular expression system, the surface display system made a significant difference in the catalytic activity of each component of the host cells (*p* < 0.05). The lower cell activity may be due to the fact that the correct display of the enzyme requires a certain period of time [28].

**Fig. 4:**
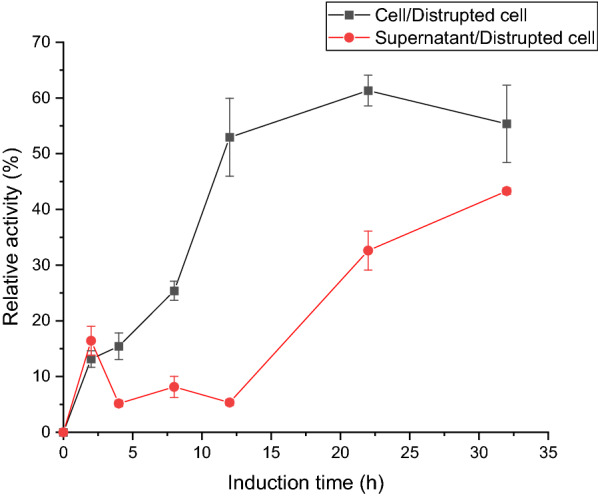
The relative activity of different fractions to the cell fragmentation. The assays were carried out in Tris–HCL buffer (pH 7.8, 50 mM) containing 20 mM KF, 1 mM SAM and induced cells or supernatant or cell debris of cell fragmentation at equal quantity for 1 h at 37 ℃. Relative values were based on catalytic ability of the disrupted cell suspension. Results are presented as the mean values and standard deviations of data from three replicates.

Thus, cells with increased induction time were used to measure changes in the catalytic ability of whole cells (Fig. 4). The ratio of the catalytic ability of the cell to the disrupted components was defined as display efficiency. As expected, the display efficiency of whole cells gradually increased with the increase of induction time. The proportion of whole-cell catalysis was stable after 20 h of induction, the result meant that the display efficiency of fluorinase reached its maximum, as reported by Li, which was about 60% [27]. This confirmed that the N-terminal of ice nucleation protein was an effective protein for surface display.

The crystal of fluorinase was resolved and displayed as a hexamer symmetrically formed by two trimers [23]. According to the crystal structure, the substrate molecule was buried in the gap between two adjacent monomers, which meant that the fusion proteins on the cell surface must be in close proximity to each other in order to bind to the substrate and achieve catalysis. There are two possible ways to generate active surface display fluorinases. The first one is that the enzymes form active units inside the cell, which then cross the cell membrane and locate on the cell surface. Another possibility is that the enzymes crosse the cell membrane in the form of monomer, then locate on the cell membrane and form active units. In this case, it is reasonable to form a single trimer on the surface of the cell due to fluidity of the membrane, but it is not for a hexamer as the movement of fluorinase in the vertical direction of the cell membrane is limited. The supernatants of cells with different induction times were analyzed by SDS-PAGE (Additional file 1), the fusion protein (INP-Faa) in the sample induced for 24 h showed a clearer band than that of 4 h of induction. Although this situation also existed in Faa cells, the overall concentration of the target protein in the supernatant was higher because all of it was accumulated inside the cells. The results showed that although the INP-Faa was mainly located on the cell membrane (Fig. 1), it will accumulate inside the cell as the culture time increases. Considering the continuous increase in the catalytic activity of the supernatant at 12–24 h (Fig. 4), we speculate that the first case is a more reasonable way to form active units. Of course, the second case is not ruled out, because it is unclear whether the transmembrane process will accompanied by depolymerization of active enzymes.

### Stability study of whole-cells

**Fig. 5:**
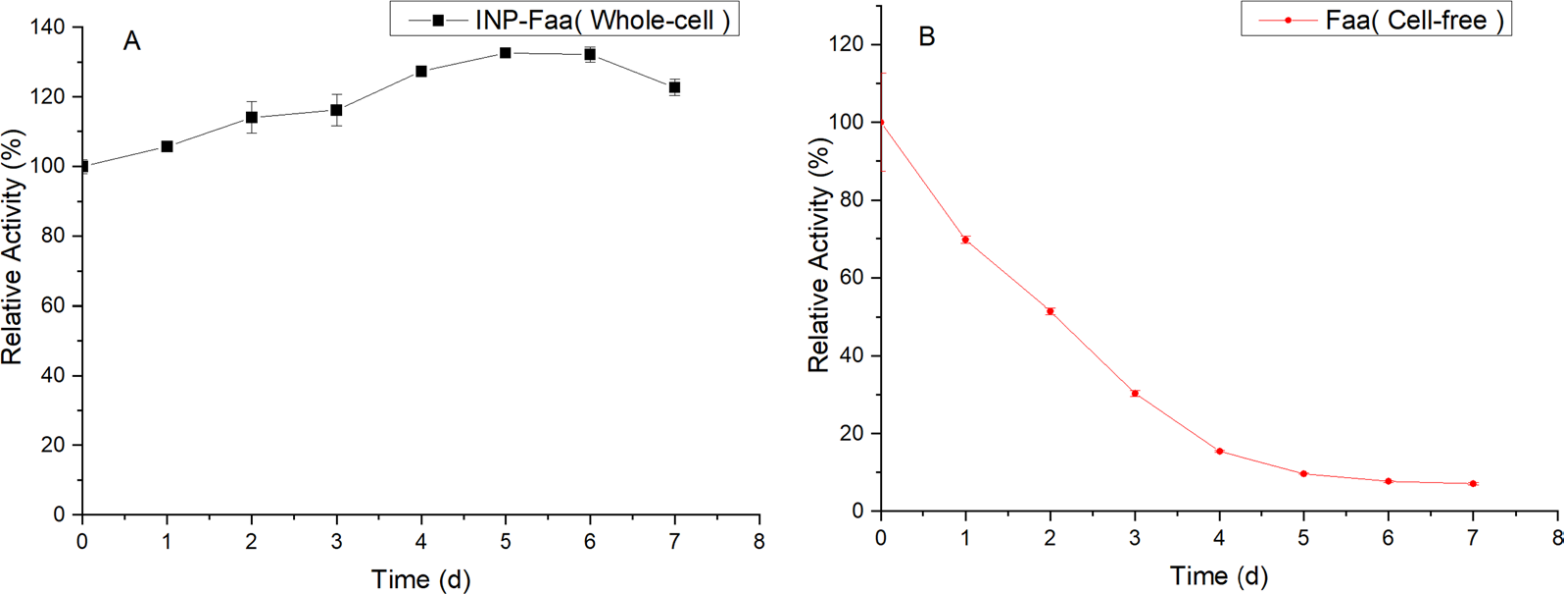
Stability of whole-cell catalyst. **A** Whole-cell of BL21(DE3)/pET28a- INP-Faa. **B** Cell free part of BL21(DE3)/pET28a -Faa. The assays were carried out in Tris–HCL buffer (pH 7.8, 50 mM) containing 200 mM KF, 1 mM SAM and 8 OD_600_ induced cells of INP-Faa or cell-free extracts of Faa at equal quantity for 2 h at 50 ℃. Relative values were based on on catalytic ability at the initial time. Each value and error bar represents the mean of three independent experiments and its standard deviation

One of the issues that cannot be ignored in biosynthesis is the stability of the enzymes. For a better comparison, the activity between the cell-free biosynthesis (Faa) and whole-cell transformation (INP-Faa) were measured for 7 days (Fig. 5). The result showed that the relative activity of INP-Faa cells kept 120% in 7-day storage at 4 ℃. In contrast, the relative activity of cell-free system was reduced by 93%. These results indicated that, compared with free enzyme, the fluorinase displayed on the cell surface had better stability.

*E. coli* contains a variety of proteases distributed in the cytoplasm, the inner membrane, and the periplasm which are responsible for the hydrolysis of abnormal and misfolded proteins [29]. The destruction of the cell membrane will lead to the contact of intracellular proteases and proteins, which may be the reason for the decrease of crude enzyme activity. The whole-cell catalyst can ensure the integrity of the membrane and prevent the degradation of enzymes. The significant increase in activity of INP-Faa may be due to the slow progress of the surface display process at low temperatures, this phenomenon has also been observed in the surface display of triphenylmethane reductase [30].

### Effect of surface-displayed fluorinase on host growth

**Fig. 6:**
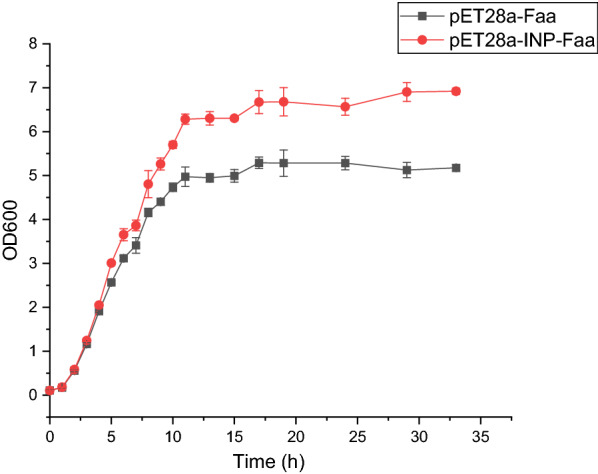
Effects of surface display systems on host growth. The cells were cultured at 37 ℃ in LB broth for about 2 h, then 0.2 mM IPTG was added for protein expression at 30 ℃. Each value and error bar represents the mean of three independent experiments and its standard deviation

The over-expression of foreign protein will cause a certain burden on the growth of the host [31]. To evaluate the influence of the surface display system on cell stability, the growth kinetics of cells carrying pET28a-Faa and pET28a-INP-Faa were compared (Fig. 6). The INP-Faa cells achieved greater cell density (OD_600_≈6.9) while Faa cells can only attain 5.2 OD_600_ after 33 h of culture. The results indicated that the surface display of foreign protein can reduce its toxicity to cells. Similar results have also been confirmed by other studies [32, 33]. More whole-cell catalysts means lower production costs, therefore, the surface display technology of fluorinase has good application prospects.

**Fig. 7:**
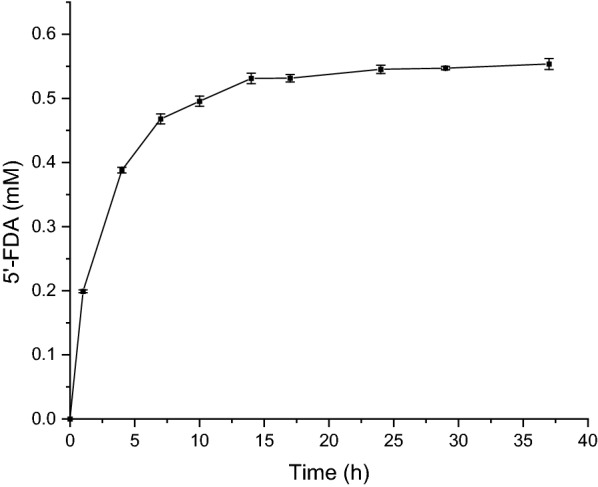
Accumulation of 5’-FDA in whole-cell catalysis. The assays were carried out in Tris–HCL buffer (pH 7.8, 50 mM) containing 200 mM KF, 1 mM SAM and 5 OD_600_ induced cells of INP-Faa at 37 ℃. Each value and error bar represents the mean of three independent experiments and its standard deviation

### Synthesis of 5’-FDA by INP-Faa-Expressing Cells

For applications, whole-cell catalysis has great advantages, including recyclability and reusability, but the efficiency of substrate utilization must also be considered. 5’-FDA was synthesized by INP-Faa displayed on *E.coli* cells. During 28 h of whole-cell catalytic transformation, 1 mM SAM was fluorinated to get maximum amount of 5’-FDA (0.55 mM) at 37 h. Compared with the immobilization method reported by previously literature (49% yield), the surface display system showed a comparable yield and reached 55% [12]. The reason for the low yield may come from instability of SAM at alkaline conditions [34]. This study was first to confirm the potential of surface display technology in whole-cell fluorination catalysis (Fig. 7).

## Conclusion

The cell surface display system provides a new height for whole-cell catalysis. In this study, the fluorinase mutant from *Amycolatopsis sp.* CA-128772 was displayed on the surface of *E.coli* by the N-terminal of ice nucleation protein. The fusion protein showed fluorinase activity and high stability. In addition, the 5’-FDA was synthesized by the bacteria-displaying fluorinase without enzyme-extracting and purifying. Furthermore, the surface display system reduced the pressure of cell growth, this meant that a large amount of whole-cell catalyst can be obtained in large-scale applications.

## Materials and methods

### Materials

Isopropyl β-D-thiogalactopyranoside (IPTG), 5′-fluorodeoxyadenosine (5′-FDA), S-adenosyl-L-methionine (SAM), KF were obtained from commercial corporations (Aladdin, Sigma-Aldrich, Macklin, etc.). The mutant sequence of fluorinase named Faa was selected from our previous studies, which showed improved catalytic efficiency. The *E. coli* strain BL21(DE3) was used as the host cell, and the ice nucleation protein (NCBI accession number: ACB59244.1) from *Pseudomonas borealis* was used as carrier protein. Plasmid pET28a was used as the expression vector.

### Construction of a fluorinase expression plasmid on the *E. coli* cell surface

The optimized gene sequence of ice nucleation protein was synthesized by the Tsingke Biotechnology Co. with pET28a between the NcoI and BamHI restriction sites to created pET28a-INP. The gene corresponding to the Faa was obtained by PCR using the following pair of primers: Faa-F 5’-ggatcttccagagatgagctcATGGCGAAACCTAGCCGC-3’ (the underline denotes SacI recognition sites) and Faa-R 5’-ctgccgttcgacgataagcttACGCTGCAACAACGCGAA-3’ (the underline denotes HindIII recognition sites). Then the PCR product was digested by SacI and HindIII restriction enzyme and connected to pET28a with the corresponding sites to form pET28a-INP-Faa. The plasmid was transformed into *E. coli* BL21 (DE3), and the recombinant strain was screened by colony PCR using primers T7 (5'-TAATACGACTCACTATAGGG-3') and T7t (5'-GCTAGTTATTGCTCAGCGG-3'). The control stain harboring pET28-Faa was also created.

### Location of fluorinase in pET28a-INP-Faa-Expressing Cells

The recombinant strain with pET28a-INP-Faa was cultivated in LB broth containing 50 µg ml^−1^ kanamycin at 37 ℃. IPTG was added at a final concentration of 0.2 mM for 4 h of induced expression at 30 ℃ when the OD_600_ reached 0.6–0.8. The cells were harvested at 6000 rpm for 10 min at 4 ℃ and washed three times with Tris–HCL (pH 7.8, 50 mM).

To demonstrate location of INP-Faa, harvested cells were fractionated to obtain cytoplasm and membrane fractions according to the method proposed by Jochen [35]. The cells were resuspended in PBS buffer (pH 8.0, 50 mM) to set OD_600_ as 5.0 and then crushed by constant cell disruption systems (Constant Systems) at 30 Kpsi. The suspension was centrifuged at 6,000 rpm for 10 min to remove undisrupted cells and large cell debris. The clarified extract was then centrifuged at 34,500 rpm for 1 h (Himac CP100WX, Hitachi, Japan) to obtain proteins from the periplasm and cytoplasm. The insoluble part was suspended in PBS containing MgCL_2_ (0.01 mM) and Triton X-100 (2%) and incubated at 25 ℃ for 30 min to dissolving inner membrane, and then the resuspended components was centrifuged at 34,500 rpm for 1 h to get the outer membranes which was insoluble. The different fractionated samples were mixed with protein loading buffer and boiled for 2 min, then the mixture was determined by 10% SDS-PAGE. *E. coli* harboring pET28a-Faa was used as a reference.

### Immunofluorescence microscopy

The INP-Faa with a his-tag was constructed for Immunofluorescence microscopy (Additional file 1). The *E. coli* cells displaying fluorinase were harvested by centrifugation at 3800 rpm at 4 ℃ for 10 min, washed three times with Tris–HCL (pH 7.8, 50 mM) and fixed in 2% formaldehyde for 10 min at room temperature. The fixed cells were washed and blocked in PBS buffer containing 2% BSA at 4 ℃ for 12 h, then his-tag antibody (Beyotime Biotechnology) was added (1:100) for another 4 h of incubation at room temperature. Alexa Fluor 555-labeled Donkey Anti-Mouse IgG (H + L) was incubated for 4 h with the cells after 3 washes. Finally, the cells were washed and examined by fluorescence microscope. Intracellular expression of Faa labeled with his-tag was used as the control group.

### Whole-cell assay

In order to verify the activity of the INP-Faa displayed on the surface, Faa was set as the control group. The induced cells were resuspended in Tris–HCL (pH 7.8, 50 mM) and divide into two equal parts, one of which was crushed. Then the disrupted cell suspension was divided into supernatant and precipitate by 6000 rpm centrifugation at 4 ℃. Subsequently, the precipitate was resuspended with an equal amount of buffer. Equal amount of cell suspension, supernatant and precipitation suspension was mixed with 20 mM KF and 1 mM SAM and incubated at 37 ℃ for 1 h. The samples were detected by HPLC after termination of the reaction by boiling for 2 min. The differences in the catalytic ability of *E. coli* cells with INP-Faa with different induction times were also compared.

### Stability study of whole-cells

The induced cells (OD_600_ = 10) containing pET28a-INP-Faa were stored in Tris–HCL (pH 7.8, 50 mM) at 4 ℃ for 7 days, and 800 µL of samples were removed each day to mix with 200 µL of substrate mixture containing 1 M KF and 5 mM SAM for 2 h incubation at 50 ℃. To verify the difference in stability between immobilized cells and crude enzyme, the assays of Faa were also carried out using the cell-free extracts.

### Effect of surface-displayed fluorinase on host growth

The cells harboring pET28a-INP-Faa or pET28a -Faa were grown in 50 mL LB broth with 50 µg ml-1 kanamycin at 200 rpm at 37 ℃ until the OD_600_ reached 0.6, and then 0.2 mM IPTG was added for protein expression at 30 ℃. The optical density of the cultures was monitored until both cultures reached the stagnate phase.

### Synthesis of 5’-FDA by INP-Faa-Expressing Cells

The reaction to investigate the ability to synthesize 5’-FDA by INP-Faa whole-cell system was carried out in Tris–HCL buffer (pH 7.8, 50 mM) containing KF (200 mM), SAM (1 mM) and cells (OD_600_ = 5) at 37 ℃. The generation of 5’-FDA was detected. The cells with pET28a-INP-Faa was induced for 24 h.

## Supplementary Information

**Additional file 1: Table S1**. The elution procedure of HPLC. **Figure S1**. The HPLC traces of the whole cell biotransformation. **Figure S2**. The SDS-PAGE of cytosol at different time points of induction.

## Data Availability

The datasets used and/or analyzed during the current study are available from the corresponding author on reasonable request.

## Abbreviations

SAM: S-adenosyl-L-methionine
5′-FDA: 5′-Fluorodeoxyadenosine
IPTG: Isopropyl-β-D thiogalactopyranoside
SDS-PAGE: Sodium dodecylsulphate polyacrylamide gel electrophoresis

## References

[1] O'Hagan D (2008). Understanding organofluorine chemistry. An introduction to the C-F bond. Chem Soc Rev.

[2] Wu YJ, Davis CD, Dworetzky S, Fitzpatrick WC, Harden D, He H, Knox RJ, Newton AE, Philip T, Polson C (2003). Fluorine substitution can block CYP3A4 metabolism-dependent inhibition: identification of (S)-N-[1-(4-fluoro-3-morpholin-4-ylphenyl)ethyl]-3-(4-fluorophenyl)acrylamide as an orally bioavailable KCNQ2 opener devoid of CYP3A4 metabolism-dependent inhibition. J Med Chem.

[3] Smart BE (2001). Fluorine substituent effects (on bioactivity). J Fluorine Chem.

[4] Charvillat T, Bernardelli P, Daumas M, Pannecoucke X, Ferey V, Besset T (2021). Hydrogenation of fluorinated molecules: an overview. Chem Soc Rev.

[5] Shimizu M, Hiyama T (2004). Modern synthetic methods for fluorine-substituted target molecules. Angew Chem Int Ed Engl.

[6] O'Hagan D, Schaffrath C, Cobb SL, Hamilton JT, Murphy CD (2002). Biochemistry: biosynthesis of an organofluorine molecule. Nature.

[7] Lowe PT, Dall'Angello S, Mulder-Krieger T, IJzerman AP, Zanda M, O'Hagan D. A New Class of Fluorinated A2A Adenosine Receptor Agonist with Application to Last-Step Enzymatic [(18) F]Fluorination for PET Imaging. Chembiochem 2017; 18(21):2156–2164. 10.1002/cbic.201700382 28851015

[8] Martarello L, Schaffrath C, Deng H, Gee AD, Lockhart A, O'Hagan D (2003). The first enzymatic method for C-F-18 bond formation: the synthesis of 5 '-[F-18]-fluoro-5 ' deoxyadenosine for imaging with PET. J Labelled Compd Rad.

[9] Tu CH, Zhou J, Peng L, Man SL, Ma L (2020). Self-assembled nano-aggregates of fluorinases demonstrate enhanced enzymatic activity, thermostability and reusability. Biomater Sci-Uk.

[10] Sun H, Zhao H, Ang EL (2018). A coupled chlorinase-fluorinase system with a high efficiency of trans-halogenation and a shared substrate tolerance. Chem Commun.

[11] Sun HH, Yeo WL, Lim YH, Chew XY, Smith DJ, Xue B, Chan KP, Robinson RC, Robins EG, Zhao HM (2016). Directed evolution of a fluorinase for improved fluorination efficiency with a non-native substrate. Angew Chem Int Edit.

[12] Sergeev ME, Morgia F, Javed MR, Doi M, Keng PY (2013). Polymer-immobilized fluorinase: recyclable catalyst for fluorination reactions. J Mol Catal B-Enzym.

[13] Li NN, Hu BJ, Wang AM, Li HMM, Yin YC, Mao TY, Xie T (2020). Facile bioinspired preparation of fluorinase@fluoridated hydroxyapatite nanoflowers for the biosynthesis of 5 '-fluorodeoxy adenosine. Sustain Basel.

[14] Driskell LO, Tucker AM, Winkler HH, Wood DO (2005). Rickettsial metK-encoded methionine adenosyltransferase expression in an *Escherichia coli* metK deletion strain. J Bacteriol.

[15] Stockbridge RB, Lim HH, Otten R, Williams C, Shane T, Weinberg Z, Miller C (2012). Fluoride resistance and transport by riboswitch-controlled CLC antiporters. Proc Natl Acad Sci U S A.

[16] Markakis K, Lowe PT, Davison-Gates L, O'Hagan D, Rosser SJ, Elfick A (2020). An Engineered *E. coli* Strain for Direct in Vivo Fluorination. ChemBioChem.

[17] Baker JL, Sudarsan N, Weinberg Z, Roth A, Stockbridge RB, Breaker RR (2012). Widespread genetic switches and toxicity resistance proteins for fluoride. Science.

[18] Urbar-Ulloa J, Montano-Silva P, Ramirez-Pelayo AS, Fernandez-Castillo E, Amaya-Delgado L, Rodriguez-Garay B, Verdin J (2019). Cell surface display of proteins on filamentous fungi. Appl Microbiol Biotechnol.

[19] Jing KJ, Guo YL, Ng IS (2019). Antigen-43-mediated surface display revealed in *Escherichia coli* by different fusion sites and proteins. Bioresour Bioprocess.

[20] Lin P, Yuan HB, Du JK, Liu KQ, Liu HL, Wang TF (2020). Progress in research and application development of surface display technology using Bacillus subtilis spores. Appl Microbiol Biot.

[21] Yuan YC, Bai XL, Liu YM, Tang XY, Yuan H, Liao X (2021). Ligand fishing based on cell surface display of enzymes for inhibitor screening. Anal Chim Acta.

[22] Gustavsson M, Muraleedharan MN, Larsson G (2014). Surface expression of omega-transaminase in *Escherichia coli*. Appl Environ Microbiol.

[23] Dong CJ, Huang FL, Deng H, Schaffrath C, Spencer JB, O'Hagan D, Naismith JH (2004). Crystal structure and mechanism of a bacterial fluorinating enzyme. Nature.

[24] Vanderveer TL, Choi J, Miao D, Walker VK (2014). Expression and localization of an ice nucleating protein from a soil bacterium, Pseudomonas borealis. Cryobiology.

[25] Liang B, Li L, Tang X, Lang Q, Wang H, Li F, Shi J, Shen W, Palchetti I, Mascini M (2013). Microbial surface display of glucose dehydrogenase for amperometric glucose biosensor. Biosens Bioelectron.

[26] Schüürmann J, Quehl P, Festel G, Jose J (2014). Bacterial whole-cell biocatalysts by surface display of enzymes: toward industrial application. Appl Microbiol Biotechnol.

[27] Li L, Kang DG, Cha HJ (2004). Functional display of foreign protein on surface of *Escherichia coli* using N-terminal domain of ice nucleation protein. Biotechnol Bioeng.

[28] Shimazu M, Mulchandani A, Chen W (2001). Simultaneous degradation of organophosphorus pesticides and p-nitrophenol by a genetically engineered Moraxella sp with surface-expressed organophosphorus hydrolase. Biotechnol Bioeng.

[29] Gottesman S (1996). Proteases and their targets in *Escherichia coli*. Annu Rev Genet.

[30] Gao F, Ding HT, Feng Z, Liu DF, Zhao YH (2014). Functional display of triphenylmethane reductase for dye removal on the surface of *Escherichia coli* using N-terminal domain of ice nucleation protein. Bioresource Technol.

[31] Palomares LA, Estrada-Mondaca S, Ramirez OT (2004). Production of recombinant proteins: challenges and solutions. Methods Mol Biol.

[32] Liang B, Li L, Mascin M, Liu AH (2012). Construction of xylose dehydrogenase displayed on the surface of bacteria using ice nucleation protein for sensitive D-xylose detection. Anal Chem.

[33] Wee MYJ, AbdMurad AM, AbuBakar FD, Low KO, Illias RM (2019). Expression of xylanase on *Escherichia coli* using a truncated ice nucleation protein of Erwinia ananas (InaA). Process Biochem.

[34] Hoffman JL (1986). Chromatographic Analysis of the chiral and covalent instability of S-adenosyl-L-methionine. Biochemistry-Us.

[35] Maurer J, Jose J, Meyer TF (1997). Autodisplay: one-component system for efficient surface display and release of soluble recombinant proteins from *Escherichia coli*. J Bacteriol.

