# Supplementary material for: Whole-cell catalysis by surface display of fluorinase on Escherichia coli using N-terminal domain of ice nucleation protein
Source: Microb Cell Fact. 2021 Oct 29;20:206. doi: 10.1186/s12934-021-01697-x (PMC8555313; doi:10.1186/s12934-021-01697-x)
Supplement: Supplementary file 1 — Additional file 1: Table S1. The elution procedure of HPLC. Figure S1. The HPLC traces of the whole cell biotransformation. Figure S2. The SDS-PAGE of cytosol at different time points of induction. [file 12934_2021_1697_MOESM1_ESM.docx]

**The optimized sequence of N-terminal of ice nucleation protein**

ATGAACGATGATAAAGTGCTGGTGCTGCGCACATGTGCAAATAATATGGCAGATCATTGCGGTCAGATCTGGCCAGTGAGCGGTGTTGTGGAATGCAAATATTGGGAACCGACCCGCAAACTGGAAAATGGCCTGGCAGGTCTGCTGTGGGGTAAAGGTGCAAGCACGCATCTGAATATGCAGGCAGATGCCCGTTGGGTTATTTGTGAAGTTGCGGTTAGCGATATTATTTTTCTGGATGCCCAGGGTGGTGTAAAATTTCCGCGTGCAGAAGTTGTTCATGTTGGTACACGTAATAGCGCAGCAGGTTATATTAGTGCAAATATTGCGAGTTATGCAAGCAGCACAGTTGCACTGAATGAAACATTTGTTTTTCCGGAAGTTCGTACCGAAACCAAAGTTGATTTTCCTGCCTCACCGGCAACAGCCGATTCAACCTTTGATATTGATCGTCATGCAACAATCCAGGGTCCTCAGACACTGGAAACCGCAGTT

**The sequence of Faa**

ATGGCGAAACCTAGCCGCCCGATTATTGCGTTTATGAGCGATCTGGGCATTACCGATGATAGCGTGGCGCAATGCAAAGGCCTGATGTTAAGCGTGTGCCCGGATGTGACCATTGTGGATGTGTGCCATACCATGAAACCGTGGGATGTGGAAGAAGGCGCGCGCTATATTGTTGATCTGCCGCGCTTATTTCCTGAAGGCACCGTGTTTGCGgctACCACCTATCCTGCGACCGGTACCACTACTCGTTCAGTTGCGCTGCGCATTAAACAGGCGGCGAAAGGTGGTGCACGTGGTCAATGGGCAGGTAGCGGTGCGGGTTTTGAACGTGCGGAAGGCAGCTATATTTATATTGCGCCGAACAACGGCTTACTGACCAGCGTGATTGAAGAACATGGCTATGTGGAAGCGTATGAAGTGAGCAGCACCGAAGTGATTCCGGAACAGCCGGAACCGACCTTTTATAGCCGCGAAATGGTGGCATTACCGgctGCGCATTTAGCAGCGGGCTTTCCTCTGGAAAAAGTGGGTCGCCCTCTGGCGGATGATGAAATTGTGCGCTTTGAACGCGCAAAACCGGCGCAAAACGATGATGGCGAACTGGTGGGTGTTGTGACCGCGATTGATCATCCGTTTGGCAACGTGTGGACCAACATTCATCGCGAAGATCTGGAAAAACTGGGCGCGGGCTATGGTACTCGTCTGCGCATTACCCTGGATGAAGTGCTGCCGTTTGATCTGCCTCTGAGCCCTACTTTTGCAGATGCGGGCCCTATTGGTACTCCTGTGGCGTATTTAAGCAGCCGCGGCTATTTAGCGCTGGCACGTAATGCGGCGAGCTTAGCGTATCCGTATAACCTGAACGCGGGCATTAGCGTTCGCGTTGTTGCAGCG

**Construction of expression plasmid of INP-Faa liganded with a his-tag**

Target gene sequence was obtained by PCR with the following pair of primers: Faa-F and Faa-RH 5’- ctcgagtgcggccgcaagcttCGCTGCAACAACGCGAAC-3’ (the underline denotes HindIII recognition sites), and then ligated to a digested pET28a-INP by SacI and HindIII using a Basic Seamless Cloning and Assembly Kit (TransGen Biotech). The INP-Faa liganded with a his-tag was used for Immunofluorescence microscopy.

**Analytical Conditions**

5’-FDA was detected by HPLC using a Poroshell 120 EC-C18 column (2.1mm×50mm, 2.7µm, Agilent) at 260 nm with gradient elution of acetonitrile and water at 0.2 mL/min.

**Table S1. The elution procedure of HPLC**

| Time (min) | Mobile phase A: Acetonitrile |
| --- | --- |
| 0 | 5% |
| 0.6 | 5% |
| 4.6 | 30% |
| 5 | 30% |
| 6 | 5% |

**Figure S1. The HPLC traces of the whole cell biotransformation**

**Figure S2. The SDS-PAGE of cytosol at different time points of induction**


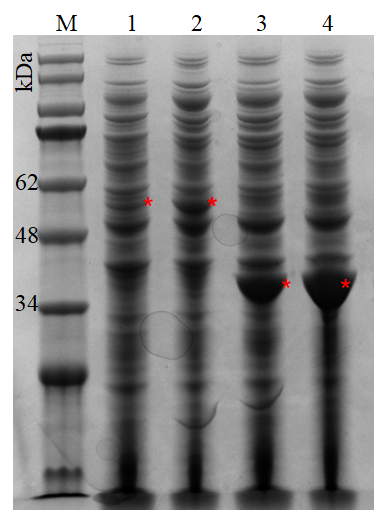


Note: Lane 1: INP-Faa of 4 hours of induction time. Lane 2: INP-Faa of 24 hours of induction time. Lane 3: Faa of 4 hours of induction time. Lane 4: Faa of 24 hours of induction time. The corresponding target protein was marked in red.
